# Immediate effects and duration of a short and single application of transcutaneous auricular vagus nerve stimulation on P300 event related potential

**DOI:** 10.3389/fnins.2023.1096865

**Published:** 2023-03-27

**Authors:** Iñaki G. Gurtubay, Diego R. Perez-Rodriguez, Enrique Fernandez, Julian Librero-Lopez, David Calvo, Pedro Bermejo, Carolina Pinin-Osorio, Miguel Lopez

**Affiliations:** ^1^Department of Neurophysiology, University Hospital of Navarre, Pamplona, Spain; ^2^Navarrabiomed Biomedical Research Centre, Pamplona, Spain; ^3^Xana Smart Neurostimulation, Epalinges, Switzerland; ^4^Arrhythmia Unit, Cardiovascular Institute, Hospital Clínico San Carlos, Madrid, Asturias, Spain; ^5^Neurologist, Translational Medicine UCB Pharma, Brussels, Belgium; ^6^Central University Hospital of Asturia, Oviedo, Spain

**Keywords:** auricular vagus nerve stimulation, event-related potentials, healthy volunteers, locus coeruleus, norepinephrine, neurostimulation, P300, transcutaneous vagus nerve stimulation

## Abstract

**Introduction:**

Transcutaneous auricular vagus nerve stimulation (taVNS) is a neuromodulatory technique that stimulates the auricular branch of the vagus nerve. The modulation of the locus coeruleus-norepinephrine (LC-NE) network is one of the potential working mechanisms of this method. Our aims were 1-to investigate if short and single applications of taVNS can modulate the P300 cognitive event-related potential (ERP) as an indirect marker that reflects NE brain activation under control of the LC, and 2-to evaluate the duration of these changes.

**Methods:**

20 healthy volunteers executed an auditory oddball paradigm to obtain P300 and reaction time (RT) values. Then a 7 min active or sham taVNS period was initiated and simultaneously a new P300 paradigm was performed. We successively repeated the paradigm on 4 occasions with different time intervals up to 56 min after the stimulation onset.

**Results:**

During active taVNS an immediate and significant effect of increasing the amplitude and reducing the latency of P300, as well as a shortening in the RT was observed. This effect was prolonged in time up to 28 min. The values then returned to pre-stimulation levels. Sham stimulation did not generate changes.

**Discussion:**

Our results, demonstrate differential facilitating effects in a concrete time window after taVNS. Literature about the modulatory effect of taVNS over P300 ERP shows a wide spread of results. There is not a standardized system for taVNS and currently the great heterogeneity of stimulation approaches concerning targets and parameters, make it difficult to obtain conclusions about this relationship. Our study was designed optimizing several stimulation settings, such as a customized earbud stimulator, enlarged stimulating surface, simultaneous stimulation over the cymba and cavum conchae, a Delayed Biphasic Pulse Burst and current controlled stimulation that adjusted the output voltage and guaranteed the administration of a preset electrical dose. Under our stimulation conditions, targeting vagal nerve fibers via taVNS modulates the P300 in healthy participants. The optimal settings of modulatory function of taVNS on P300, and their interdependency is insufficiently studied in the literature, but our data provides several easily optimizable parameters, that will produce more robust results in future.

## 1. Introduction

The vagus nerve (VN) is involved in the regulation of multiple systems and has an important role in maintaining homeostasis. Stimulating this nerve to modulate the function of related organs has long drawn the attention of investigators.

Invasive vagus nerve stimulation (iVNS) has been approved in the last three decades as a therapeutic option in the management of drug resistant-epilepsy and treatment-resistant depression (Daban et al., 2008; Beekwilder and Beems, 2010; Yuan and Silberstein, 2016; Gadeyne et al., 2022) and is currently investigation for other conditions. However, surgical risks, technical challenges and potential side effects have limited its application (Ventureyra, 2000; Fitzgerald, 2013).

In recent years, two non-invasive transcutaneous vagus nerve stimulation (tVNS) methods have been developed and their ease of use has made it possible to extend their therapeutic effects (Kaniusas et al., 2019a,b). Superficial stimulation of the neck region, targets the same vagal fibers as in the implanted version, but requires high stimulation intensity and produces motor activation that produces discomfort. The second approach is to stimulate the auricular branch of the vagus nerve (ABVN) which innervates the outer ear (Berthoud and Neuhuber, 2000; Dabiri et al., 2020). This is referred to as transcutaneous auricular VN stimulation (taVNS). The rationale of taVNS is based on anatomical studies demonstrating that certain parts of the ear area have afferent VN distribution (Henry, 2002; Peuker and Filler, 2002; Trevizol et al., 2015; Bermejo et al., 2017). Electrical stimulation of these areas may produce activity changes in the VN pathway in the brain stem and central structures (Shiozawa et al., 2014), resulting in a modulation effect similar to iVNS (Rong et al., 2012; Hein et al., 2013; Carreno and Frazer, 2016) being safe and well tolerated (Redgrave et al., 2018). taVNS received CE-approval for epilepsy and depression in 2010, for pain in 2012 and anxiety in 2019 being an effective treatment option (Ma et al., 2022; von Wrede et al., 2022). Currently, taVNS is being studied as a therapy for chronic tinnitus (Shim et al., 2015), atrial fibrillation (Stavrakis et al., 2015, 2020), prediabetes (Huang et al., 2014), migraine (Silberstein et al., 2016), rehabilitation after ischemic stroke (Baig et al., 2019), ventricular arrhythmias (Nasi-Er et al., 2019) and respiratory symptoms associated to COVID-19 (Tornero et al., 2022) among others (Mertens et al., 2018). The potential beneficial effects of taVNS on several aspects of behavior, emotion or cognition have also been investigated in recent years (Jacobs et al., 2015; Jongkees et al., 2018; Mertens et al., 2020).

The vagus nerve terminates at the nucleus tractus solitarii (NTS) where it projects onto the locus coeruleus (LC), the main hub of noradrenaline (also referred to as norepinephrine, NE) in the cortex (Burger et al., 2020). Actual data strengthens the hypothesis that the modulation of the locus coeruleus-norepinephrine (LC-NE) network is one of the potential working mechanisms of VNS (Rodenkirch et al., 2022). The effect of VNS on LC-NE activity is well established in animals, but studies on the noradrenergic effects of (*t*) VNS in humans are lacking. Direct measurement of NE requires an invasive procedure and suffers from poor reliability and sensitivity (Grassi and Esler, 1999). Instead, the activity of the LC-NE network can be inferred from indirect markers (Burger et al., 2020) such as the P300, a non-invasively measured cognitive event related potential (ERP). The generation of the main component of P300 ERP, also known as P3 (Picton et al., 2000), depends on the LC-NE system (Nieuwenhuis et al., 2005; Polich, 2007; Brown et al., 2015; de Rover et al., 2015) and is evoked by an active attentional response to infrequent target stimuli appearing randomly interspersed in a series of non-target standard frequent stimuli. It is considered a marker that reflects higher level cognitive processes in which stimulus processing (evaluation and categorization) and response selection are involved. From a neurophysiological point of view, one of the most accepted hypotheses about the P300 is that it represents active context updating of the working memory (Polich, 2007) serving as a link between stimulus characteristics and attention (Näätänen, 1990). A distributed network of neuro-inhibitory processes is presumed to promote efficacious processing of relevant stimuli as reflected in an enhanced P300 (Polich, 2007), as a consequence of increased phasic activity in LC neurons in response to unpredicted target stimuli that demands immediate behavioral response (Aston-Jones and Cohen, 2005; Murphy et al., 2011). Thus, the amplitude of this ERP reflects the amount of attentional resources used during the task (Helm et al., 2016) and its latency reflects how long it takes to categorize the target stimulus as relevant, i.e., it indicates mental processing speed (Polich, 2007; Yarkoni et al., 2009; Martinez Vargas, 2014; Hajcak and Foti, 2020; Verleger, 2020).

To date, it has been shown that in healthy subjects taVNS generates an immediate modulatory effect with an increase in the amplitude of the P300 in auditory (Rufener et al., 2018) or visual (Ventura-Bort et al., 2018) tasks. This change is similar to those recorded during iVNS (Ventura-Bort et al., 2018; Sharon et al., 2021), neck VNS (Lewine et al., 2019) or pharmacological interventions aimed at stimulating the NE pathway (Sanfins et al., 2017). Other immediate effects have also been reported such as the reduction in the P300 latency and reduction in the reaction time response (Fischer et al., 2018; Rufener et al., 2018). These findings raise the possibility of modulating selective attention and improving the stimulus processing speed through taVNS. Nevertheless, further studies are needed to clarify the consistency of these effects, because, on the one hand negative or partial results have also been reported for some of these topics (Fischer et al., 2018; Warren et al., 2019; Gadeyne et al., 2022), and on the other, the duration of these effects have not been systematically studied.

Given the important role of the LC-NE system in the mechanism of action of taVNS and the link between the P300 and the NE system, we investigated the modulatory effect of a single and short (7 min) application of taVNS stimulation and/or sham (ineffective) stimulation, compared to basal situation, on the latency and amplitude parameters of the P300 ERP. In addition, accuracy and RT were assessed as indicators for potential cognitive effects on behavioral levels. The temporal variation of these changes were evaluated by repeating blocks of P300 in different time windows from 0 to 56 min after the taVNS onset.

## 2. Materials and methods

### 2.1. Study population

A total of 26 healthy volunteers participated in the present study, approved by the ethics committee of our hospital (210623/PI_2021-79). Healthy volunteers, aged between 18 and 39 years old, were included. Recruitment took place among postgraduate students’ tertiary level education. A narrow range in both age and education level was chosen to keep the population as homogeneous as possible (Picton et al., 2000). To obtain a balanced sample, we aimed for a similar number of men and women.

First, a full explanation of the procedure was given and signed informed consent was obtained from each subject. Then a demographic questionnaire about the medical history and lifestyle of the participant was completed. Exclusion criteria were (1) acute or chronic intake of any medication or recreational drug, (2) history of neurosurgical treatment, (3) ear lesions or infections, (4) hearing disorders, (5) allergy to Ag/AgCl or to platinum, (6) pregnancy or breastfeeding, and (7) history of traumatic brain injury.

Twenty-six healthy volunteers gave informed consent, of whom one was excluded. On the remaining 25 subjects, only 20 participated in the test in active condition (mean age 29.7, 10M:10F), and of these, 10 also participated in sham condition (mean age 27.7, 4M:6F). All patients were selected randomly. An overview of all included subject’s characteristics can be found in Table 1.

**Table 1:** Demographic and threshold data.

Condition	*N*	Gender M/F	Age (years)		St (mA)		Dt (mA)	
			Mean (SD)	Range	Mean (SD)	Range	Mean (SD)	Range
taVNS	20	10/10	29.7 (5.4)	18–39	0.48 (0.11)	0.3–0.6	3.82 (0.24)	3.3–4
Sham	10	4/6	27.7 (5.7)	18–36	0.47 (0.09)	0.3–0.6	3.83 (0.26)	3.3–4

Mean, standard deviation (SD) and range. M, male; F, female; St, sensory threshold; Dt, discomfort threshold; mA, milliampere.

Many external factors such as the circadian and ultradian rhythm, recent food intake, caffeine, alcohol or drugs, may affect arousal levels and are known to modulate the P300 ERP (Polich and Kok, 1995). To minimize their effects variations in conditions between subjects were kept to a minimum. The experiments were carried out from July to September. All sessions took place between 14:00 and 16:00 in a controlled temperature room and with artificial light source. The subjects were instructed to have a light digestible lunch 2 h before the experiment. Participants were asked not to consume alcohol, any energy drink or caffeine-containing beverages 24 h prior to the experiment.

### 2.2. Stimulation conditions

**Figure 1:**
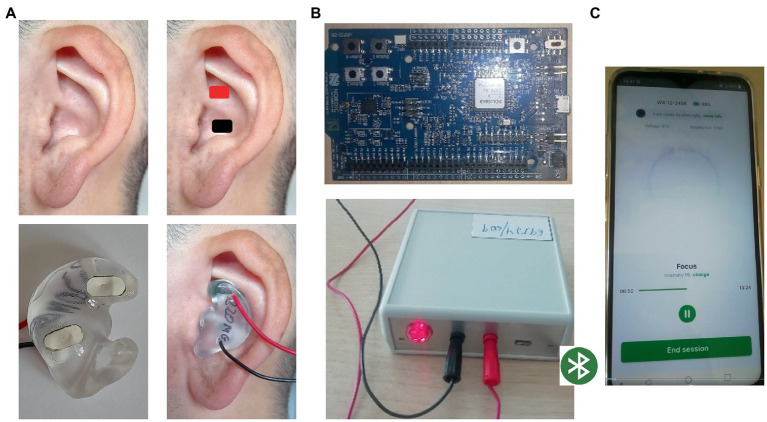
taVNS system. **(A)** Customized auricular device with stimulating platinum electrodes over cymba and cavum conchae physically connected to B; **(B)** electrical stimulus generator connected by Bluetooth to C; **(C)** stimulation control module in a smart phone app. That allowed to control the stimulating parameters.

**Figure 2:**
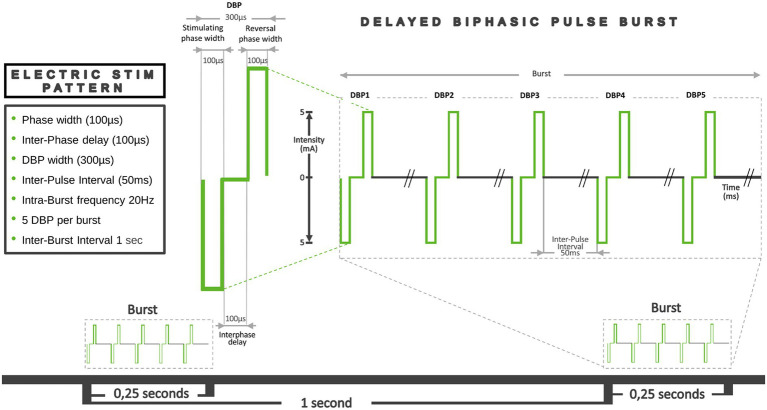
Graphical representation of the main parameters of the DBP-type burst pacing pattern. Pulse width: the duration of a single electrical pulse. In this case, we used Delayed Biphasic Pulse (DBP), which consisted of three consecutive distinct periods: an initial 100 μs stimulation period, an intermediate 100 μs interphase delay phase and a final reversal phase 100 μs stimulation period. So, a total DBP duration was 300 μs. The time between each of these pulses or Inter-Pulse Interval (IPI) was 50 ms. The number of pulses in a train, their duration and the IPI determined the intra-train frequency (20 Hz). Total duration of each train was 0.2515 s. Thus, 5 pulses were applied every quarter of a second, which means a frequency of around 20 Hz. The Inter-Burst Interval (IBI) defined as the time that passes between one train and the next, was 1 s.

Our taVNS system consists of three parts. The auricular stimulation device was anatomically customized for each volunteer. An audiologist made a mold of the left auricle (outer ear) using Otoform^®^Ak kneadable impression material from Dreve Otoplastik Gmbh (Unna, Germany). After a 3D scan, a customized earbud was manufactured. The device had two integrated round squared 8 × 4 mm platinum electrodes with a surface area of 30 mm^2^, located in the cymba and cavum conchae, and was connected to an electrical stimulus generator *via* a standard cable. Figure 1, shows the position of the stimulation electrodes. The electrical stimulus generator was an external portable unit. For the present test, a train-pacing pattern with delayed biphasic pulses (DBP) was used. Figure 2 shows the main parameters and a schematic view of DBP-type burst. This pattern is equivalent to pacing at a frequency of 20 Hz and a pulse width of 300 μs. The electrical generator was connected by Bluetooth to a smart phone app. That worked as a stimulation control module.

In order to homogenize the stimulation parameters between the subjects, and to guarantee the delivery of an equal electrical dose, a current controlled stimulation system was used. First, a specific intensity value was set for stimulation period, which conditioned the desired effectively applied electrical total dose that was to be administered at the end of the aforementioned period. During the stimulation, the auricular device continuously measured the impedance between the skin and the stimulation electrodes. The data was then sent to the control module where, by means of software and depending on the impedance values and the time remaining to the end of stimulation, continuously adjusted the output voltage to ensure the delivery of the preset current dose. The app. Stored these variables and also allowed their visualization on the smartphone display during the test. As recommended (Farmer et al., 2021) in order to avoid intensity peaks or undesired effects, the maximum intensity was limited to 5 mA in the app, far from the accepted safety threshold of 10 mA (Kraus et al., 2013; Lamb et al., 2017), and was later adjusted to the subject’s Pt.

During the active condition, we set an initial stimulation value of 1.9 mA, resulting in a total of 798 μC to be delivered over 7 min to all subjects. These values were calculated based on our previous experience (Garcia de Gurtubay et al., 2021) and preliminary test. To check that the intensity value was adequate and comfortable, before the start of the experimental procedure, we measured the sensitive (St) and discomfort threshold (Dt) for each volunteer increasing the current, from 0 to 5 mA, in steps of 0.1 mA. Two conditions had to be met in order to continue with the experiment: the pre-selected stimulation value of 1.9 mA must be at least 2.5 times higher than the sensitive threshold and 20% below the discomfort threshold value. All subjects met those conditions. Threshold values are shown in Table 1.

During the sham condition, we maintained the stimulation device in the same topographic area. We measured St and Dt and we stimulated for 7 min with an intensity 0.1 mA below the sensitive threshold of each subject. In this condition, the mean stimulation intensity was 0.37 mA (155 μC), range 0.2–0.5 (84–210 μC), SD 0.09. Threshold values are shown in Table 1.

**Figure 3:**
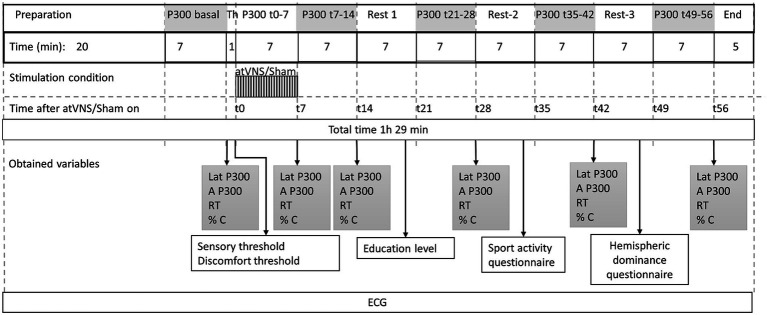
Stimulating protocol sequence. 12 consecutive periods with their respective duration are shown. Time 0, was considered at the beginning of taVNS or sham stimulation. Variables obtained along the periods are shown. Time (*t*) in minutes. Latency (Lat), Amplitude (A), Reaction time (RT), accuracy (% C) % of target sounds correctly indicated, Th, Threshold; ECG, electrocardiogram.

The protocol, was divided into 12 periods or blocks, and was timed by a stopwatch. For the active condition, the sequence was as follows (Figure 3): First a preparation period of around 20 min. Then, the subjects executed an oddball task of 7 min in length, to obtain P300 basal values. The electric stimulation earbud was positioned and St and Dt were measured. Following this, active taVNS was initiated and a new P300 block was performed simultaneously. Using the time related to the stimulation onset, we called it P300 t0-7. Immediately after we removed the earbud, and a new P300 block was performed (P300 t7-14), followed by a 7 min rest period. We then repeated P300 blocks followed by rest periods to obtain P300 t21-28, P300 t35-42 and P300 t49-56. The final electrode removal period lasted 5 min. The protocol had a total duration of 1 h and 29 min, and had to be completed in full for the results to be considered as valid.

For sham condition, we used exactly the same sequence. The only modification was the reduction of stimulus intensity during P300 t0-7 period to sub-umbral levels.

In the rest periods between the P300 blocks, subjects were allowed to drink some water and/or go to the bathroom and active conversation was maintained. During these periods, other data was collected, such as the level of education, sports activity data and a hemispheric dominance questionnaire (Veale, 2014), to be analyzed in case of finding differential effects between individuals.

The design of the study was single-blind. When active and sham conditions had to be executed, they were randomized in a balanced order, and defined as “different types of stimulation” rendering the participants unaware of the fact that there was a real and sham taVNS stimulation condition. Both conditions were completed on separate days, with a time window of 2–3 weeks.

### 2.3. Eliciting the P300. Auditory oddball paradigm

P300 ERP were elicited by carrying out a two-stimulus auditory oddball task (Picton et al., 2000; Duncan et al., 2009) in which subjects, while sited, were instructed to push a predefined button as quickly as possible with their dominant hand in response to 1,000 Hz target tones, while ignoring 500 Hz non-target standard tones. Auditory stimulation was administered binaurally using Nicolet Unshielded 300 Ohm headphones (TDH39) for medical use. Sound intensity was set at 70 dB SPL. Tones had a duration of 100 ms including 5 ms of rise and fall phases. They were presented every 1 s (1 Hz), in a non-stop block of 7 min, consisting of a random sequence of 84 target and 336 standard tones, with a probability of occurrence of 0.2: 0.8. During the preparation period, a test run of 15 stimuli was completed to familiarize themselves with the different tones.

Subjects remained in an enclosed area, free from noise and other distractions. To avoid eye movement during the task, they were asked to focus their gaze on a fixed point 1 meter away. In addition, the eye movements were monitored by vertical electro-oculogram (EOG) recording using two Ag/AgCl cup-electrodes (Ambu^®^ Neuroline 726 20M/10) located on the superior and inferior region of the left orbit.

The subject response button, allowed to obtain reaction time (RT) and accuracy in percentage of the correct responses to target stimuli. This system of RT measurement does not influence the results of the evoked potentials and is considered a useful tool to evaluate the amount of attentional resources used to detect targets during the task (Wagenmakers and Brown, 2007; Lempke et al., 2020).

Stimulus presentation and recording of RT were managed with Synergy version 22.3.0.21 from Natus^®^ 2019 (Middleton, WI, United States).

### 2.4. Electrophysiological recordings

The recording procedure was carried out in accordance with the guidelines and recommended methods for obtaining ERP in clinical research (Picton et al., 2000; Duncan et al., 2009).

The electro-encephalogram (EEG) was recorded using disposable 1 cm diameter Ag/AgCl cup electrodes (Ambu^®^ Neuroline 726 20M/10) positioned on Fz, Cz, Pz, Oz, A1 and A2 sites of the international 10–20 system. AFz was used as a ground electrode. A2 was used as online reference with offline averaging with A1. They were filled with Ten20^®^ conductive paste and electrode impedance, measured before all the P300 blocks, was kept below 8 KΩ.

Signal acquisition was recorded with Synergy version 22.3.0.21 from Natus^®^ system, online digitized with a sampling rate of 512 Hz, with bandpass filters 0.01–100 Hz. The EEG signal epoch was 1,000 ms, including 100 ms period prior to the auditory stimulus. The automatic signal rejection system was set at ≥100 μV, so that when these signals were exceeded, they were automatically excluded from the recording.

Natus system acquires and automatically averages all recording signals to show an averaged ERP but does not allow the individual signal processing. For this reason, during online recording, individual sweeps were acquired and stored using CED signal software version 5.12a (CED^®^ Cambridge, United Kingdom). Then, signals were processed offline and electrophysiological ERP analysis was conducted using Matlab version 9.2 R2017a (Mathworks, MA, United States) on a Hp Laptop (Palo alto, CA, United States).

taVNS has been shown to be a safe procedure, but because of the potential modulatory effect on cardiac function (Kreuzer et al., 2012; Stavrakis et al., 2015), we performed electrocardiographic (ECG) monitoring throughout the whole procedure. A Nuubo^®^ (Smart solutions technologies, SL, Madrid, Spain) ECG monitoring system with wireless textile-based wearable containing ECG sensors was used (Perez de Isla et al., 2011; Olmos et al., 2014). 2 ECG leads were recorded and analyzed offline.

### 2.5. Measurement of the P300 ERP

First, the EEG-signals or trials were visually inspected for major artifacts (slow fluctuations or high frequency signals) and rejected if necessary. We also excluded trials with incorrect behavioral responses, that is, when no response was found to a target sound and those in which the response was located 150 ms before the tone (considered anticipation) or 700 ms after the tone (considered failure).

Independent component analysis (ICA) was then directly applied to the data in order to subtract artifact components from each electrode. Next, linked mastoid re-referencing, band-pass filtering (0.1–30 Hz) and baseline correction on the 100 ms pre-stimulus interval were performed. Finally, trials were averaged separately for standard and target stimuli, for each condition, participant and block.

For target stimuli, a minimum of 90% accuracy of correct responses and at least 74 valid trials were required to average and accept the ERP as valid. P300 isolation was accomplished through creation of a target-standard difference wave and defined as the largest positive deflection on the parietal midline electrode Pz, between 250 and 600 ms after stimulus onset. The correct detection of P300 peak was verified visually on each plot. The P300 peak amplitude (μV) measurement was done relative to the prestimulus baseline. P300 latency (ms) was measured as the position in time from stimulus onset to the point of maximum positive amplitude within specified time window (Picton et al., 2000; Polich, 2007; Duncan et al., 2009). As the focus of the present study was to describe the changes induced by the taVNS on the P300 component over time, only this ERP is reported.

### 2.6. Statistics

All statistical analyses were conducted with R version 4.2.0 (R Core team, 2022, Vienna, Austria). The level of statistical significance was set at 0.05. Behavioral and electrophysiological data were analyzed using repeated measures ANOVA and linear mixed regression models (LMM). In these last analyses, measurement time, exposure status-plus the interaction between them-were introduced as fixed effects and individuals as a random intercept.

## 3. Results

### 3.1. Electrical tolerance and cardiovascular effects

Electrical stimulation was well tolerated and all subjects completed the protocol. No adverse effects (vegetative, headache, pain, dizziness or skin irritation; Ellrich, 2011; Mertens et al., 2018; Redgrave et al., 2018; Kaniusas et al., 2019a) were triggered.

In terms of cardiovascular safety, no clinically relevant events were reported. ECG lead analysis did not show abnormalities in the heart rate and/or arrhythmias (sustained bradycardia, sustained tachycardia, extra-beats episodes).

### 3.2. Behavioral results

The behavioral and electrophysiological results are summarized in Table 2.

**Table 2:** Behavioral and electrophysiological results (mean values with their standard error) along the repeated blocks in active and sham condition are shown.

Condition	P300 blocks						
	Mean (SD)	Basal	t0–7	t7–14	t21–28	t35–42	t49–56
Active	Accuracy (%)	97.1 (1)	96.6 (1.4)	97.1 (1.3)	97.1 (1.1)	97.5 (0.3)	96.8 (1.4)
	RT (s)	0.336 (0.078)	0.304 (0.079)	0.290 (0.074)	0.310 (0.075)	0.332 (0.076)	0.337 (1.4)
	A (μV)	10.98 (3.48)	15.59 (4.6)	19.68 (5.3)	16.39 (5.19)	12.14 (4.1)	11.04 (3.4)
	Lat (ms)	346.25 (23)	321.55 (23.3)	310.9 (24.1)	325.95 (23.1)	338.3 (24.1)	341.1 (23.2)
sham	Accuracy (%)	97.5 (1.4)	97.4 (1.8)	97.2 (1.3)	97.2 (0.8)	97.8 (1)	97.7 (1.3)
	RT (s)	0.346 (0.065)	0.347 (0.065)	0.346 (0.078)	0.348 (0.069)	0.352 (0.07)	0.350 (0.063)
	A (μV)	10.53 (2.24)	10.79 (2.42)	10.47 (2.26)	10.33 (2.24)	10.57 (2.2)	10.5 (2.2)
	Lat (ms)	348.1 (14.88)	342.2 (13.47)	346.3 (13.2)	347.4 (15.6)	347.3 (15.1)	346.8 (17.6)

Accuracy: percentage of correctly detected targets. RT, reaction time; s, seconds; A, P300 amplitude; μV, microvolts; Lat, P300 latency; ms, milliseconds.

The mean accuracy over the repeated blocks ranged from 96.6% to 97.8%. No significant effects were observed between blocks or conditions.

**Figure 4:**
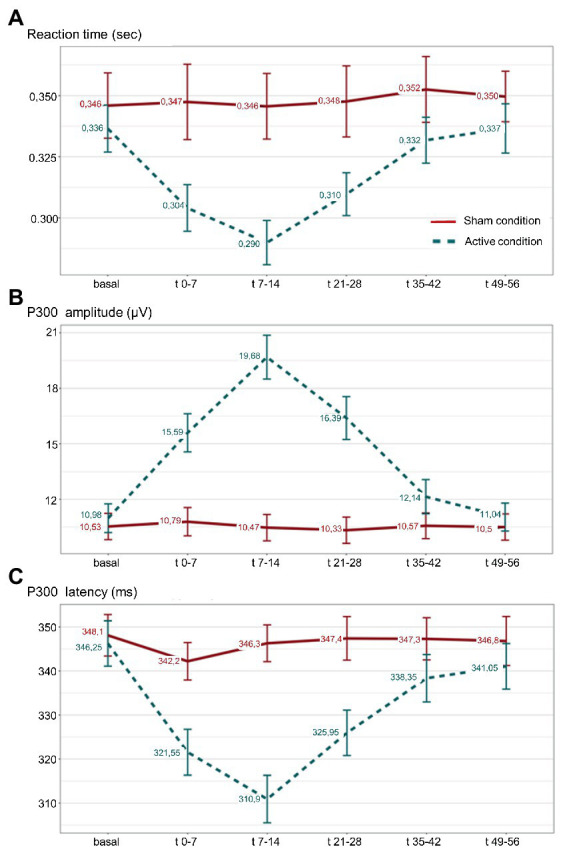
**(A)** Reaction time (RT); **(B)** amplitude; and **(C)** P300 latency, mean values along successive P300 blocks during active taVNS (dashed line) and sham (continuous line) conditions are represented, with their respective standard error of the mean. Sec: seconds; μV: microvolts; ms: milliseconds;

The analysis of RT showed a significant effect during active taVNS condition compared to the same period of sham taVNS condition, being significantly shorter in *t*(0–7), *t*(7–14) and *t*(21–28) time windows. LMM analysis confirmed the differences in aforementioned time windows (*p* < 0.001), showing that RT was on average 0.034 s, 0.046 s, and 0.028 s shorter than expected in t0-7, t7-14, and in t21-28 blocks, respectively. No differences were found in basal or t35-42 and t49-56 attempts. See Supplementary Table 3. No gender differences were observed. Figure 4A, represents the evolution of mean RT, and standard error of the mean (SEM) intervals, along successive P300 blocks during sham and active taVNS.

### 3.3. Electrophysiological results

Table 2 shows the mean P300 amplitude and latency values along the repeated blocks in taVNS and sham conditions.

Analysis of P300 amplitude showed a significant effect during active taVNS condition compared to the same period of sham taVNS condition, being significantly higher in t0-7, t7-14 and t21-28 blocks. Linear mixed regression models confirmed the differences (*p* < 0.001), showing that P300 amplitude was on average 4.35 μV, 8.76 μV, and 5.61 μV higher than expected in t0-7, t7-14 and t21-28 blocks. See Supplementary Table 4. No differences were found in basal or t35-42 and t49-56 attempts. No gender differences were observed. Figure 4B, represents the evolution of mean P300 amplitude, and SEM intervals, along successive P300 blocks during sham and active taVNS conditions.

The analysis of P300 latency revealed a significant effect during active taVNS condition compared to the same period of sham taVNS condition, being significantly shorter in t0-7, t7-14 and t21-28 blocks. LMM analysis confirmed the differences (*p* < 0.001), showing that P300 latency was on average 18.80 ms, 33.55 ms and 19.60 ms shorter than expected in aforementioned time periods. See Supplementary Table 5. No differences were found in basal or t35-42 and t49-56 attempts. No gender differences were observed. Figure 4C, represents the evolution of mean P300 latency, and SEM intervals, along successive P300 attempts during sham and active taVNS conditions.

## 4. Discussion

Under our experimental conditions, we found that 7 min of taVNS induced changes in the P300 ERP parameters (amplitude increase and latency decrease) and a shortening in the RT. These effects occurred rapidly upon taVNS onset and lasted at least up to t21-28 min time window. All values then returned to pre-stimulation levels. This relationship was consistent for all volunteers.

The amplitude of the P300 can be used as a marker for taVNS-induced phasic LC-NE network activity, reflecting modulatory changes that documents potentially beneficial effects of stimulating the vagus nerve on specific aspects of behavior, cognition and memory (Aston-Jones and Cohen, 2005; Murphy et al., 2011; Burger et al., 2020). According to this theory, taVNS activates the LC-NE system and stimulates NE ascending projections to cortical structures (Aston-Jones and Cohen, 2005). Due to the widespread release of NE to the neocortex, taVNS also increases the NE release in the fronto-parietal attention network. The generation of the principal component of P300 ERP, depends mainly on the LC-NE system (Nieuwenhuis et al., 2005), although dopaminergic and cholinergic systems are also involved (Warren et al., 2019). This excitatory effects of taVNS on LC activity and the NE transmitter system, and the close relationship between P300 and LC-NE system, provides causal evidence for the LC-P3 hypothesis (Rufener et al., 2018), which postulates that the P300 amplitude is the electrophysiological manifestation of the NE-modulated increase in phasic arousal to task relevant stimuli and reflects attention allocation and stimulus evaluation. According to this theory the P300 should also be closely associated with the speed and accuracy of the response. As is described in this section, the P300 literature is consistent with this hypothesis. Our findings, of active taVNS induced P300 amplitude increase are also consistent with results from pharmacological interventions showing that the application of NE-agonist increases this ERP (Brown et al., 2015; Rufener et al., 2018).

The literature review shows positive effect of taVNS on the P300 amplitude of healthy subjects with amplitude increase during auditory (Rufener et al., 2018) or visual paradigms (Ventura-Bort et al., 2018) but also lack of significant effects during auditory (Gadeyne et al., 2022) or visual paradigms (Fischer et al., 2018; Warren et al., 2019). If these studies fail to find an effect, it is unclear whether this is due to a failure to activate the VN (Hagen et al., 2014) or whether successful stimulation does not affect the process under investigation, relative to an active control condition (Burger et al., 2020). The current guidelines for eliciting, recording, and quantifying the P300 ERP in clinical research provides a standardized methodology and facilitates comparability and interpretation (Picton et al., 2000; Duncan et al., 2009), however there is not an standardized system for taVNS and currently the great heterogeneity of stimulation approaches concerning targets and parameters, make it difficult to obtain conclusions about this relationship (Farmer et al., 2021). Otherwise, NTS mediated effects on the LC may be excitatory, inhibitory or neutral (Vonck et al., 2014) depending on the posology and timing of the taVNS and could also explain some of the negative results.

In addition to the expected P300 amplitude changes, we have also found a modulatory effect of taVNS with a reduction in P300 latency and a shortening in the RT response. Rufener et al. (2018) using auditory P300 task reported a P300 latency reduction after taVNS, a parameter that is thought to reflect the duration of stimulus evaluation, that is, how long it takes to categorize the target stimulus as relevant, i.e., it indicates mental processing speed (Polich, 2007; Yarkoni et al., 2009; Martinez Vargas, 2014; Hajcak and Foti, 2020; Verleger, 2020). This finding indicates that taVNS accelerated the reorienting toward the target stimulus and the respective evaluation process in the oddball paradigm. A comparable facilitating effect was found in a study that assessed the effect of taVNS in an action selection task (Steenbergen et al., 2015). Our results also showed a taVNS related P300 latency reduction, in good agreement with the assumption that taVNS shapes the general arousal of the attention network, suggesting that neural resources relevant for stimulus discrimination were optimized.

With regard to VNS effects over RT data during P300 ERP studies, according to LC-P3 hypothesis an improvement on speed of responding could be expected. We have to point out that this parameter has been poorly analyzed. An RT reduction has been reported in epileptic patients receiving chronic iVNS during the auditory oddball paradigm (De Taeye et al., 2014). Using taVNS during visual task, RT reductions have been reported, either descriptively (Warren et al., 2020) or statistically (Fischer et al., 2018) as a direct effect of taVNS on behavioral measures of conflict-triggered recruitment of cognitive control. Our RT data shortened significatively, immediately from the initial stimulation period to the t21–28m time period, and then returned to pre-stimulation values in subsequent attempts, which is likely to be a direct effect of stimulation rather than a learning effect after repeated exposure to the target stimulus along the blocks. Research on the relationship between RT and P300 indicates that in a simple oddball task, RT occurs about 50 ms before the peak of the P300 ERP (Picton, 1992) and P300 latency correlates with RT (Trejo et al., 1991) as observed in the present experimental conditions.

Taken as a whole, our results, P3 amplitude increase, and P3 latency and RT shortening, in a concrete time window after taVNS, demonstrate differential facilitating effects of taVNS, enhancing the certainty of the subject to discriminate between standard and target stimuli and to identify the latter one as relevant to initiate a behavioral reaction improving the auditory selective attention.

In our opinion, the strength of the results is due to the fact that the study was designed after a detailed analysis that attempted to avoid the technical and protocol difficulties described in other experiments. Based on the literature review and our experience we will discuss our findings and specifically address aspects to be considered regarding stimulation protocol and parameters that could influence the effect of taVNS, giving practical tips to optimize the stimulation settings in taVNS.

### 4.1. Stimulation location

The specific ear region selected for stimulation is based on sparse and limited anatomical studies of human auricle dissection (Fay, 1927; Tekdemir et al., 1998; Peuker and Filler, 2002), which indicate that the cymba conchae and cavum, as well as the external auditory channel as having the highest density areas of ABVN projections. It is known that the cymba conchae of the external ear is innervated exclusively by this branch, but other regions of the external ear receive important afferent innervation by ABVN solely or shared with other nerves such as the cavity of the conchae (Berthoud and Neuhuber, 2000) the tragus, and the posterior and inferior walls of the ear canal (Peuker and Filler, 2002; Bermejo et al., 2017).

Several studies support the stimulation of most of these areas mainly cymba conchae, cavum and/or the inner wall of the tragus, recording the postsynaptic brainstem activity from the VN nuclei registered at the scalp as far field potential and called vagus somatosensory evoked potential (vSEP) (Fallgatter et al., 2003; Polak et al., 2009; Lewine et al., 2019) by measuring the activation of NTS and LC pathway on functional MRI (Yakunina et al., 2017). According to these findings, one of the most widely used commercial taVNS system (Frangos et al., 2015; Burger et al., 2018; Yap et al., 2020) NEMOS (distributed by tVNS Technologies, formerly Cerbomed), stimulates over cymba conchae (Kaniusas et al., 2019a) but other targets have also been used such as the cavum (Ay et al., 2015), the tragus (Badran et al., 2017), the external auditory channel or some of its specific parts (Kraus et al., 2013; Farmer et al., 2021). Recently, simultaneous stimulation over the cymba and cavum region of the ear has been shown to produce better vSEP response than cymba-conchae stimulation alone (Garcia de Gurtubay et al., 2021) being an easily optimized parameter.

### 4.2. The electrode-tissue interface

The electrode-tissue interface is an often-neglected aspect in taVNS literature. Skin impedance and properties of subcutaneous tissue affect the current flow. Adequate skin cleaning and degreasing before stimulation can easily reduce skin-level impedances and increase the current that reaches nerve fibers (Badran et al., 2019).

Materials used as electrodes for charge injection must meet several requirements such as being biocompatible, mechanically acceptable for the application and effective. During electrical stimulation faradic reactions should not occur at levels that are toxic to the surrounding tissue nor cause premature failure of the electrode, (Merrill et al., 2005). The most commonly used stimulation electrodes for the ear are made of titanium (Yap et al., 2020), although other different materials have been used such as Ag/AgCl, pure silver, … (Farmer et al., 2021). We chose platinum for our taVNS electrodes because its electrical and mechanical characteristics (Merrill et al., 2005) met all our requirements. Some published studies (Fang et al., 2016; Yakunina et al., 2018) and systematic reviews (Sigrist et al., 2022), offer insufficient information on the materials or even the size of used electrodes, which is another limitation for our collective understanding of the electrode-tissue interface and its interactions.

A small stimulation surface (5.3 mm^2^), such as that used by the NEMOS device applies the electrical charge over a small area and thus reduces the tolerance and the Dt. In an electrode size comparative study, it was concluded that the use of larger electrodes (54 mm^2^) improves the stimulation efficiency, produces greater activation of the NTS at lower intensities, and makes the subject’s perception of the electrical stimuli more comfortable, allowing the distribution of the electrical charge over a greater surface and decreasing the intensity of the charge (Garcia de Gurtubay et al., 2021). This evidence is extremely important because modulatory effects require repetitive taVNS sessions lasting from minutes to hours. With this in mind, the stimulation surface cannot be extremely large because it could leads to stimulation of other nerve fibers or muscular activation and produce confounding effects. In this study, we have used medium sized electrodes (30 mm^2^) which in our experience, have all the advantages of greater ones allowing for a more topographically restricted stimulation.

**Figure 5:**
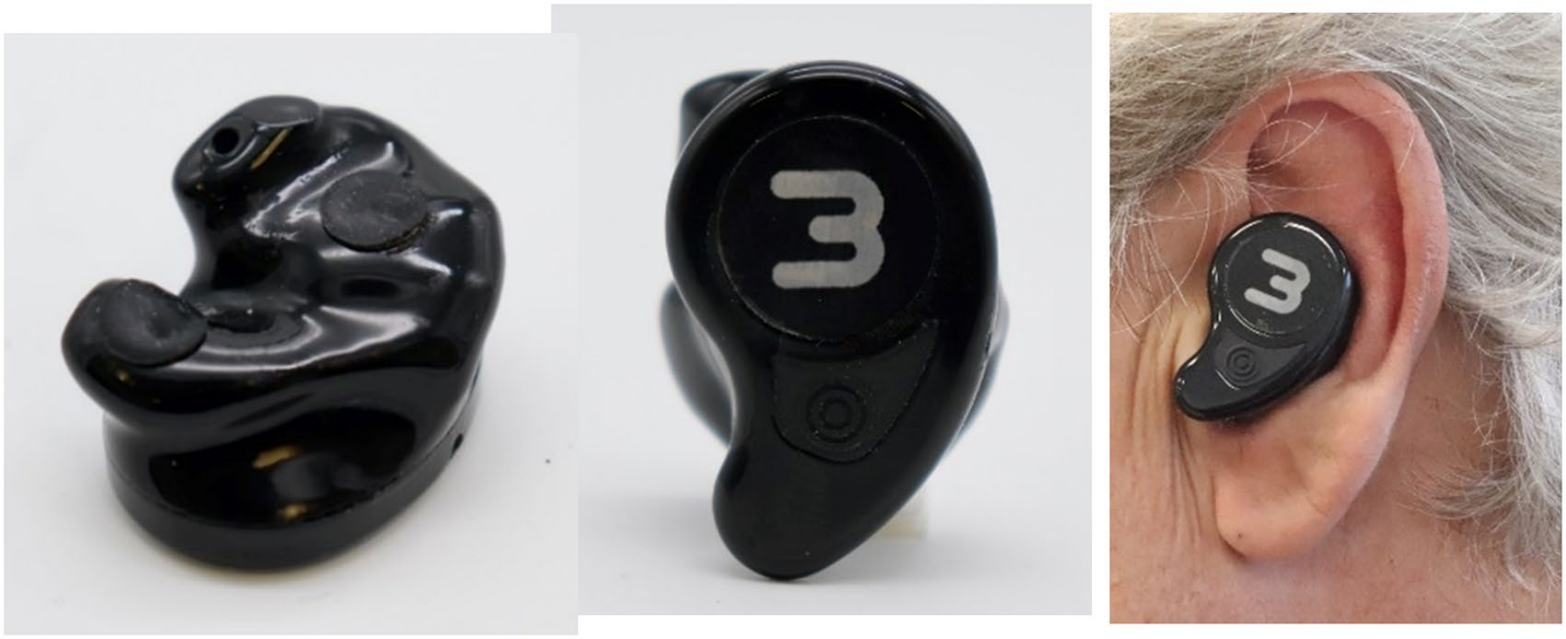
Wireless taVNS device. Image courtesy of Xana^®^ Smart Neurostimulation.

A correct stimulation electrode fit is a crucial factor in maintaining adequate contact in the electrode-tissue interface with low impedance during the stimulation period. Several approaches have been used for taVNS. Initially, bracket electrodes attached to the ear lobe or the tragus were used. They are easy to use, but high pressure of the brackets on the ear can cause discomfort, skin irritation, swelling and inflammation (Bolz and Bolz, 2022). The NEMOS device initially focused in stimulating the tragus, and the electrode applicator of this device consisted of a silicone ring placed between the tragus and the antitragus and used a plastic spacer to position the electrode directly into the cymba conchae. This configuration provided rather poor horizontal fixation to the stimulating electrode and partially blocked the external ear canal. Ear clips (Lehtimäki et al., 2013; Liu et al., 2018) and headsets (Hein et al., 2013) have also been used. Recently a headphone-like applicator has been designed to improve the positioning and fixation of the NEMOS device (Bolz and Bolz, 2022). In our opinion due to the great anatomical variability of the ear, the use of one-size-fits-all design is not a correct methodological strategy, at least when experimental research tries to clarify fundamentals on taVNS such a possible modulatory effect over P300. The use of these electrode types (Rufener et al., 2018; Warren et al., 2019; Gadeyne et al., 2022), is likely to limit our understanding on these interactions, regardless of positive or negative results. In such situations, a motion free attachment of standard stimulating electrodes must be achieved to avoid movement. We suggest using single flexible or adaptable electrodes with conductive adhesive (Garcia de Gurtubay et al., 2021), or using a personalized stimulation device such as that used in this paper. Using this approach during our research we have not found any non-responders. In any case, all these electrodes will be penalized by the gravitational effect of the wire, which is very sensitive to movement. The solution in the future should come from wireless electrodes (Figure 5).

### 4.3. Stimulation intensity

The ultimate goal of stimulation is to deliver a dose of electrical charge that is sufficient to produce the desired physiological changes. The stimulation intensity must be sufficient to stimulate ABVN, that is predominantly innervated by the large myelinated A-fibers (Safi et al., 2016), and generate nerve potentials on the NTS as the first step to spread the effect to higher levels. Low stimulation intensities fail in activation of NTS. Tolerance is considered to decrease above 8 mA (Aihua et al., 2014; Fang et al., 2017) and higher intensities, over 10 mA, generate discomfort, pain, motor activation and other undesired responses (Woodbury and Woodbury, 1991; Kaniusas et al., 2019a).

In terms of pacing intensity, most taVNS use intensities in the range of 0.2 up to 8 milliampere (mA) (Rong et al., 2012; Hein et al., 2013; Mollet et al., 2013; Aihua et al., 2014; Trevizol et al., 2015; Liu et al., 2017; Loerwald et al., 2018; Gurtubay et al., 2020; Garcia de Gurtubay et al., 2021), even somewhat higher (Lamb et al., 2017).

Currently, there are no human studies that have systematically investigated the effect of different stimulation intensities for taVNS (Ludwig et al., 2021) but animal and theoretical data could help on this topic. Studies conducted in anesthetized dogs have shown a stimulation threshold for vagus large myelinated A-fibers around 0.4 mA (Yoo et al., 2013), and giving the similarities in the fiber thickness between canine and human vagal nerves, it has been postulated that human vagus nerves could follow the same pattern. Furthermore, based on histological examination and using computational models it has been estimated that, with pulse widths between 200 and 500 μs, intensities between 0.75 and 1.75 mA are sufficient to cause vagal activation (Helmers et al., 2012).

According to previous data, low intensities with stimulation output current of 0.5 mA × 35 min (Warren et al., 2019) or 0.6 mA × 17 min (range 0.2–1.4; Gadeyne et al., 2022) could explain some negative results in the modulation of the P300 ERP by taVNS, being insufficient to adequately modulate the LC. On the other hand, Rufenner (Rufener et al., 2018) found positive effect with amplitude increase and latency decrease by applying atVNS with the same device at 0.5 mA × 100 min and simultaneously transcranial random noise stimulation (tRNS) over the frontal cortex. We could speculate that the low stimulation intensity may be compensated by the long stimulation time or even by tRNS that is supposed to have a potentiation-like effect over cortical excitability, probably due to the modulation of sodium channels, but more studies are need to clarify these effects.

Different approaches can be used to overcome under stimulation. Some studies titrate stimulus intensity to the participant’s perceptual (sensory) threshold (Badran et al., 2018) but evidence shows that is not enough. A common approach is to set an empirical intensity value, above the sensory threshold and below the discomfort threshold that is administered for a specific period of time. Other authors use tingling but non painful sensation of stimulation to achieve maximum effect (Ventura-Bort et al., 2018; Széles et al., 2021). A practical approach, consisting of measuring St, and applying a stimulation intensity from at least 2.5 or 3 times higher than St. to 15–20% under Dt values, is usually used in clinical practice to generate somatosensory evoked potentials (Aminoff and Eisen, 1999) and guarantee adequate stimulation of myelinated A-fibers. Our experience using this method in previous (Garcia de Gurtubay et al., 2021) and current research support the generation of an adequate vagal response, with intensities in the range of literature recommended and far from pain thresholds or intensities that are considered uncomfortable.

The dose response relationship has been poorly tested in humans. Using sensory evoked potentials it was shown that with increasing stimulation intensity in the neck (Nonis et al., 2017) or ear (Polak et al., 2009) an increase in vSEP was achieved. In rats, it has been demonstrated that VNS leads to an intensity-dependent increase in brain NE in response to stimulation of the left vagus nerve (Roosevelt et al., 2006; Raedt et al., 2011). Animal research (Roosevelt et al., 2006) showed that stimulation at a current strength of 0.0 mA and 0.25 mA did not affect the NE concentrations in the hippocampus or the cortex, while 0.5 mA stimulation significantly increased the NE concentrations bilaterally and 1.0 mA stimulation led to even higher elevated levels of NE in the hippocampus and also in the cortex (bilateral). Based on these findings we can assume that within certain stimulation range values, the concentration of NE is a function of the intensity of the VNS (Vonck et al., 2014) and more intense stimulation leads to a broader recruitment and activation of a higher number of vagal nerve fibers and causes a higher firing rate of neurons in the LC (Groves et al., 2005; Dorr and Debonnel, 2006), leading to higher concentrations of NE in the hippocampus and cortex. These NE increases in animal studies are transient and return to baseline levels when the stimulation is stopped and the vagus nerve is not being stimulated. These findings explain the immediate effects observed over P300 parameters and/or RT values in this, and other studies (Rufener et al., 2018; Ventura-Bort et al., 2018; Lewine et al., 2019).

On the other hand, the effective duration of these effects after a single short session of taVNS have not been studied. Lewine et al. after 5 min of transient neck VNS stimulation, reported P300 amplitude increases and changes over other electroencephalographic parameters that in some cases lasting >2 h post-stimulus, probably due to activation of different neural networks with the participation of different neurotransmitter systems including those for GABA, acetylcholine, serotonin and NE (Lewine et al., 2019). In the present experiment, we have found significative changes on P300 parameters and on RT lasting up to t21-28 min time window.

Greater effects of VNS in females have been previously reported in animal studies, probably because of the effect of estrogens to the muscarinic acetylcholine in the central nervous system (Du et al., 1994). It has been postulated that similar effects might be expected in human subjects due to hormonal levels (Koenig and Thayer, 2016) and differences in the neuronal pathways and neuronal sensitivity (Janner et al., 2018). We found no gender differences in our statistical analysis.

### 4.4. Stimulation posology

Stimulating taVNS settings and protocols show great heterogeneity concerning stimulation posology. Several parameters will influence the electrical output dose, affecting the reliably and effectively received charge, and so the final clinical effects. There is no standardized method that allows us to calculate and compare the amount of applied effective electric current. This is one of the most difficult and confounding factors when the efficacy of taVNS intervention is being achieved.

Applied daily doses of taVNS differ, ranging from minutes to several hours per day, in continuous or in intermittent (on–off) patterns. Similarly, applied stimulation intensities, as stated before, vary from 0.1 to 10 mA. Numerous publications support the safety of performing taVNS using frequencies of 20–30 Hz and/or pulse widths of 100–300 μs (Rong et al., 2012; Hein et al., 2013; Aihua et al., 2014; Trevizol et al., 2015; Liu et al., 2016, 2017; Fang et al., 2017; Lamb et al., 2017) with modulatory efficacy on diverse vagal functions and absolute absence of adverse effects. What is more, studies with electrical stimulation current and durations much higher and therefore much higher total charge doses, are safe and do not generate local adverse effects (Kaniusas et al., 2019a,b).

The development of invasive VNS techniques, have recently focused on optimizing the shape of the stimulation pulse and stimulation protocol (Fitchett et al., 2021) but due to the novelty of non-invasive VNS, advances on this topic are still scarce. To date, most taVNS studies employ monophasic stimulation (MS). Recently, because their electrical and physiological advantages (Bolz and Bolz, 2022) other pulse shapes have been used as biphasic stimulation (BS), triphasic stimulation (TS) or other shape variations, such as DBP type stimulation, specifically designed to optimize the activation of neural fibers, lowering the stimulation threshold and thus increasing the recruitment volume even more (Reilly and Diamant, 2011; Kaniusas et al., 2019b). These new patterns have been successfully used with modulatory purposes over different diseases such epilepsy, tinnitus and cluster headache (Hyvärinen et al., 2015; Yap et al., 2020). Studies on the efficiency of stimulation pattern have been only performed on percutaneous VNS (Kaniusas et al., 2020) and indicate that the TS pattern has clinical (*in vivo*) and experimental (in-silico models) superiority over BS which is superior to MS. To our knowledge this study is the first to use a DBP pattern to study the effect of taVNS on P300 ERP, and could be another reason for the positive effects obtained.

All these stimulation settings, including the stimulation time and the intensity at which it is performed, are those that determine the theoretical electrical dose to be administered. Depending on the impedance, this dose will differ from the effectively received dose at the end of the taVNS session. Most devices use a constant voltage approach (voltage-controlled stimulation), in which the intensity of stimulation is set at a concrete value during the whole stimulation period. This system is highly dependent on the good or poor contact between the stimulation electrode and the subject’s skin, that produces variation of impedance on the electrode-tissue interface, as this directly affects the induced current in the excitable auricular tissue generating fluctuations in the real administered voltage and strongly influences the resulting stimulation efficiency (Farmer et al., 2021). Due to this variable inter-individual and intra-individual impedance, the constant voltage approach leads to different output currents according to Ohm’s law, and that makes medical comparisons impossible. As these variables are not routinely monitored, is not possible to know the real administered dose of electrical charge. This fact, in which the received dose is well below what is theoretically expected, is possibly the most important factor of negative results.

Instead of the constant voltage approach, it might be better to use a constant current output device (current controlled stimulation), with a closed loop regulator in which the output current is measured, and the output voltage is immediately adapted to keep the current constant (Bolz and Bolz, 2022). Our device was designed in such a manner, and in addition to allowing the reception of specific and equal electrical dose for all subjects, allows the data to be stored, computing the accumulated received dose in use of the taVNS over prolonged periods and enabling in future sessions to adjust doses depending on physiological response of the user. As recently suggested (Bolz and Bolz, 2022) using electrotechnical and software-based improvements to the state-of-the-art stimulators, such evolution algorithms that use device and subject data to optimize stimulation parameters, will allow the use of individualized taVNS therapy.

## 5. Conclusion

The literature on the modulatory effect of taVNS over P300 ERP shows a wide range of results. With regard to presented data and taken together, we demonstrate taVNS related modulation over P300 parameters, as well over behavioral response. These significative changes, start immediately during the 7 min taVNS stimulation period and extend in time up to the t21-28 min time window, and then returning to baseline levels.

This study demonstrates facilitating effects of taVNS, according to the LC-NE theory and the LC-P3 hypothesis that postulates a modulatory effect of taVNS that improves some of the underlying mechanisms of auditory selective attention such as arousal level, stimulus evaluation and identification processes, and behavioral response execution.

Considering all stimulation parameters, it is evident that the optimal settings of modulatory function of taVNS on P300, and their interdependency is insufficiently studied in humans, making it difficult to draw conclusions from experimental or clinical studies. In any case, a detailed analysis of the literature and the results of the present study, has allowed us to detect easily optimizable settings, such as a customized earbud stimulator, enlarged stimulating surface, simultaneous stimulation over cymba and cavum conchae, a DBP burst and current controlled stimulation. This knowledge will produce more robust results in future applications and a better understanding of the underlying mechanisms, to further improve this technique and identify its correct positioning as a therapeutic device.

## 6. Limitations

There were several limitations associated with the study. First, we have sample differences between active condition (N20) and sham condition (N10). Our statistical analysis has considered these differences and does not produce biases in the results. Anyway further studies with larger sample sizes should be performed. Second, the study was focused on the instant modulation of P300 ERP after a short and single application of taVNS. Long-term treatment should be included in further research. Third, because the changes of cognitive potentials related to age or educational level, the extrapolation of results can only be carried out for healthy subjects, in the same age range and educational level as the group studied. Finally, we want to discuss the difference in stimulation between active condition (1.9 mA) and sham (under threshold), related to sensation *vs* no-sensation of being stimulated, and specifically the potential arousal level changes due to lack of sensation in the sham condition. Although the arousal influences throughout the procedure, its direct relationship on the observed changes is very unlikely, since they would only have occurred in t0–7 block and not in the successive ones, where there was no stimulation. In addition, during active stimulation the electrical intensity was so low that the habituation phenomenon occurred around 2 min, so that for most of the time the subjects did not perceive the electrical stimulus. Stimulation during sham with 1.9 mA intensity over lobe or another vagal inactive area would have raised doubts about the possible influence of other nerve afferents.

## Data availability statement

The raw data supporting the conclusions of this article will be made available by the authors, without undue reservation.

## Ethics statement

The studies involving human participants were reviewed and approved by the Ethics Committee of the Health Department of the Government of Navarre (ref 2021/79). The patients/participants provided their written informed consent to participate in this study.

## Author contributions

IG, EF, DC, PB, and ML contributed to conception and design of the study. IG and DP-R performed data acquisition. IG, DP-R, DC, and CP-O organized and performed data analysis. JL-L performed the statistical analysis. IG wrote the first draft of the manuscript. JL-L wrote sections of the manuscript. All authors contributed to the article and approved the submitted version.

## Conflict of interest

Xana Smart Neurostimulation, provided a B version of the taVNS device for this study IG received advisory board fees from Xana. ML is the Chief Scientific officer of Xana. EF is the Chief Executive Officer of Xana. PB is the Chief Innovation Officer and DC has become the Chief Medical officer in the late part of this study.

The remaining authors declare that the research was conducted in the absence of any commercial or financial relationship that could be construed as a potential conflict of interest.

## Publisher’s note

All claims expressed in this article are solely those of the authors and do not necessarily represent those of their affiliated organizations, or those of the publisher, the editors and the reviewers. Any product that may be evaluated in this article, or claim that may be made by its manufacturer, is not guaranteed or endorsed by the publisher.
