# Supplementary material for: Immediate effects and duration of a short and single application of transcutaneous auricular vagus nerve stimulation on P300 event related potential
Source: Front Neurosci. 2023 Mar 27;17:1096865. doi: 10.3389/fnins.2023.1096865 (PMC10083261; doi:10.3389/fnins.2023.1096865)
Supplement: Supplementary file 1 [file Data_Sheet_1.docx]

Supplementary data. Linear mixed model analysis. Tables 3, 4 and 5.

|  | **RT** | | |
| --- | --- | --- | --- |
| *Predictors* | *Estimates (sec)* | *CI* | *p* |
| (Intercept) | 0.350 | 0.331 – 0.370 | **<0.001** |
| stim [active] | -0.014 | -0.025 – -0.003 | **0.017** |
| stest [t 0-7] | 0.001 | -0.011 – 0.014 | 0.818 |
| stest [t 7-14] | -0.000 | -0.013 – 0.013 | 0.963 |
| stest [t 21-28] | 0.002 | -0.011 – 0.015 | 0.794 |
| stest [t 35-42] | 0.007 | -0.006 – 0.019 | 0.311 |
| stest [t 49-56] | 0.004 | -0.009 – 0.017 | 0.569 |
| stim [active] * stest [t 0-7] | -0.034 | -0.050 – -0.018 | **<0.001** |
| stim [active] * stest [t 7-14] | -0.046 | -0.062 – -0.031 | **<0.001** |
| stim [active] * stest [t 21-28] | -0.028 | -0.044 – -0.013 | **<0.001** |
| stim [active] * stest [t 35-42] | -0.011 | -0.027 – 0.004 | 0.155 |
| stim [active] * stest [t 49-56] | -0.004 | -0.019 – 0.012 | 0.647 |
| **Random Effects** | | | |
| σ^2^ | 0.00 | | |
| τ_00_ _id_ | 0.00 | | |
| ICC | 0.88 | | |
| N _id_ | 20 | | |
| Observations | 180 | | |
| Marginal R^2^ / Conditional R^2^ | 0.223 / 0.903 | | |

TABLE 3. Linear mixed model analysis of the Reaction Time (RT)

|  | **P300 A** | | |
| --- | --- | --- | --- |
| *Predictors* | *Estimates*  *(µV)* | *CI* | *p* |
| (Intercept) | 11.44 | 9.36 – 13.51 | **<0.001** |
| stim [active] | -0.46 | -1.93 – 1.01 | 0.539 |
| stest [t 0-7] | 0.26 | -1.39 – 1.91 | 0.756 |
| stest [t 7-14] | -0.06 | -1.71 – 1.59 | 0.943 |
| stest [t 21-28] | -0.20 | -1.85 – 1.45 | 0.811 |
| stest [t 35-42] | 0.04 | -1.61 – 1.69 | 0.962 |
| stest [t 49-56] | -0.03 | -1.68 – 1.62 | 0.971 |
| stim [active] * stest [t 0-7] | 4.35 | 2.33 – 6.38 | **<0.001** |
| stim [active] * stest [t 7-14] | 8.76 | 6.74 – 10.79 | **<0.001** |
| stim [active] * stest [t 21-28] | 5.61 | 3.59 – 7.64 | **<0.001** |
| stim [active] * stest [t 35-42] | 1.12 | -0.90 – 3.15 | 0.274 |
| stim [active] * stest [t 49-56] | 0.09 | -1.93 – 2.11 | 0.930 |
| **Random Effects** | | | |
| σ^2^ | 3.50 | | |
| τ_00_ _id_ | 14.51 | | |
| ICC | 0.81 | | |
| N _id_ | 20 | | |
| Observations | 180 | | |
| Marginal R^2^ / Conditional R^2^ | 0.326 / 0.869 | | |

Table 4. Linear mixed model analysis of the P300 amplitude (A).

|  | **P300 Lat** | | |
| --- | --- | --- | --- |
| *Predictors* | *Estimates*  *(ms)* | *CI* | *p* |
| (Intercept) | 346.31 | 335.29 – 357.33 | **<0.001** |
| stim [active] | -0.06 | -6.85 – 6.74 | 0.987 |
| stest [t 0-7] | -5.90 | -13.54 – 1.74 | 0.129 |
| stest [t 7-14] | -1.80 | -9.44 – 5.84 | 0.643 |
| stest [t 21-28] | -0.70 | -8.34 – 6.94 | 0.857 |
| stest [t 35-42] | -0.80 | -8.44 – 6.84 | 0.837 |
| stest [t 49-56] | -1.30 | -8.94 – 6.34 | 0.737 |
| stim [active] * stest [t 0-7] | -18.80 | -28.16 – -9.44 | **<0.001** |
| stim [active] * stest [t 7-14] | -33.55 | -42.91 – -24.19 | **<0.001** |
| stim [active] * stest [t 21-28] | -19.60 | -28.96 – -10.24 | **<0.001** |
| stim [active] * stest [t 35-42] | -7.10 | -16.46 – 2.26 | 0.136 |
| stim [active] * stest [t 49-56] | -3.90 | -13.26 – 5.46 | 0.412 |
| **Random Effects** | | | |
| σ^2^ | 74.96 | | |
| τ_00_ _id_ | 460.94 | | |
| ICC | 0.86 | | |
| N _id_ | 20 | | |
| Observations | 180 | | |
| Marginal R^2^ / Conditional R^2^ | 0.214 / 0.890 | | |

TABLE 5. Linear mixed model analysis of the P300 latency (Lat)
